# Progress in Prognostic Metabolic Biomarkers for Coronary Artery Disease Patients Post-Percutaneous Coronary Intervention

**DOI:** 10.31083/RCM39597

**Published:** 2025-10-27

**Authors:** Zi-Teng Zhang, Jun-Hui Li, Guo-Qing Qi, Hong-Liang Zhao

**Affiliations:** ^1^Heart Center, The First Hospital of Hebei Medical University, 050031 Shijiazhuang, Hebei, China

**Keywords:** coronary artery disease (CAD), post-PCI, metabolic indicators, early prediction, prognosis

## Abstract

Percutaneous coronary intervention (PCI) has made significant progress as one of the main treatments for coronary artery disease (CAD), but the risk of major adverse cardiovascular events (MACE) after PCI remains high. Therefore, early identification of high-risk CAD patients after PCI and improvement of risk factors are crucial for patient prognosis. Although various prognostic biomarkers related to CAD have been identified, most of them have not been widely applied in clinical practice. Recent studies have found that some simple and easily obtainable metabolic indicators have early predictive value for the prognosis of CAD patients after PCI, mainly including four categories: blood lipids and related metabolites, blood glucose and related metabolites, nutrition-related metabolites, and kidney-related metabolites. This review synthesizes the four aforementioned categories of indicators with the aim of integrating their unique characteristics to enable precise prognostication in patients after PCI, deepen mechanistic insights, and furnish evidence-based guidance for clinical decision-making.

## 1. Introduction

Coronary heart disease (CHD) is one of the leading diseases globally, with a complex pathogenesis involving multiple risk factors. To date, no single biomarker has been identified that can comprehensively assess disease severity and accurately predict prognosis. Dysregulation of metabolic indicators such as blood lipids, blood glucose, serum albumin, and uric acid is closely related to the onset, progression, and long-term prognosis of CHD. Particularly after percutaneous coronary intervention (PCI), the impact of these metabolic indicators on patient prognosis is more pronounced. For instance, elevated levels of low-density lipoprotein cholesterol (LDL-C) promote foam cell formation, increased triglyceride (TG) levels affect lipoprotein metabolism and fuel inflammatory responses, and reduced levels of high-density lipoprotein cholesterol (HDL-C) can further exacerbate atherosclerosis. Additionally, insulin resistance (IR) can accelerate CHD progression by promoting inflammation, oxidative stress, and dyslipidemia. Other metabolic indicators, such as low serum albumin (SA) levels, may reflect chronic inflammation and malnutrition, while elevated serum uric acid (SUA) levels can lead to increased oxidative stress, endothelial dysfunction, and enhanced inflammatory responses, thereby promoting the development of atherosclerosis [1, 2, 3, 4, 5]. Lipid indices are directly linked to long-term plaque stability [6]. Glycemic indices reflect metabolic control [7]. Albumin and uric acid–related indices primarily indicate acute-phase stress and microcirculatory status, conferring greater sensitivity for predicting short-term complications such as length of stay and collateral circulation [8, 9, 10]. Therefore, strict control of blood lipids, blood glucose, and uric acid levels is crucial for improving the prognosis of patients after PCI.

Epidemiological evidence indicates that mortality remains high and prognosis unfavorable despite timely and aggressive PCI [11]. Moreover, unplanned readmissions after PCI impose a substantial burden on healthcare systems and are attracting increasing attention [6]. Consequently, there is a pressing need to develop effective prognostic biomarkers to predict and optimize post-procedural management [12, 13]. This review synthesizes several categories of metabolic indicators that offer early insight into post-PCI outcomes. It aims to integrate their distinctive characteristics and elucidate underlying mechanisms. Additionally, it provides an evidence-based reference for clinical decision-making.

## 2. Lipid and Related Metabolic Indicators

### 2.1 LDL-C

A study has established a causal relationship between LDL-C and coronary artery disease (CAD) [14]. Despite many CAD patients achieving target LDL-C levels with statin therapy, the incidence of major adverse cardiovascular events (MACE) remains high [15]. This indicates that solely reducing LDL-C levels is insufficient to fully prevent MACE, especially after PCI.

### 2.2 LDL-C Cumulative Exposure

Research indicates that the role of LDL-C in cardiovascular disease risk is not only related to its current level but also to its cumulative exposure over time. Previous studies, often focusing on single-time-point LDL-C measurements (usually in middle and old age), underestimate the impact of long-term cumulative effects. A cohort study found that the cumulative exposure to LDL-C (calculated as age × LDL-C) has a stronger correlation with increased CAD risk than single measurements of LDL-C [16]. However, the relationship between LDL-C cumulative exposure and atherosclerosis progression is not fully understood [17], and studies on its predictive value for post-PCI patient prognosis are limited. Future multicenter, prospective studies with larger sample sizes stratified by age should combine optical coherence tomography (OCT) findings with coronary angiography. This approach will help assess the relationship between LDL-C cumulative exposure and the extent of atherosclerotic progression, as well as its prognostic value in patients after PCI.

### 2.3 Lipoprotein (a) [Lp(a)]

**Fig. 1.**
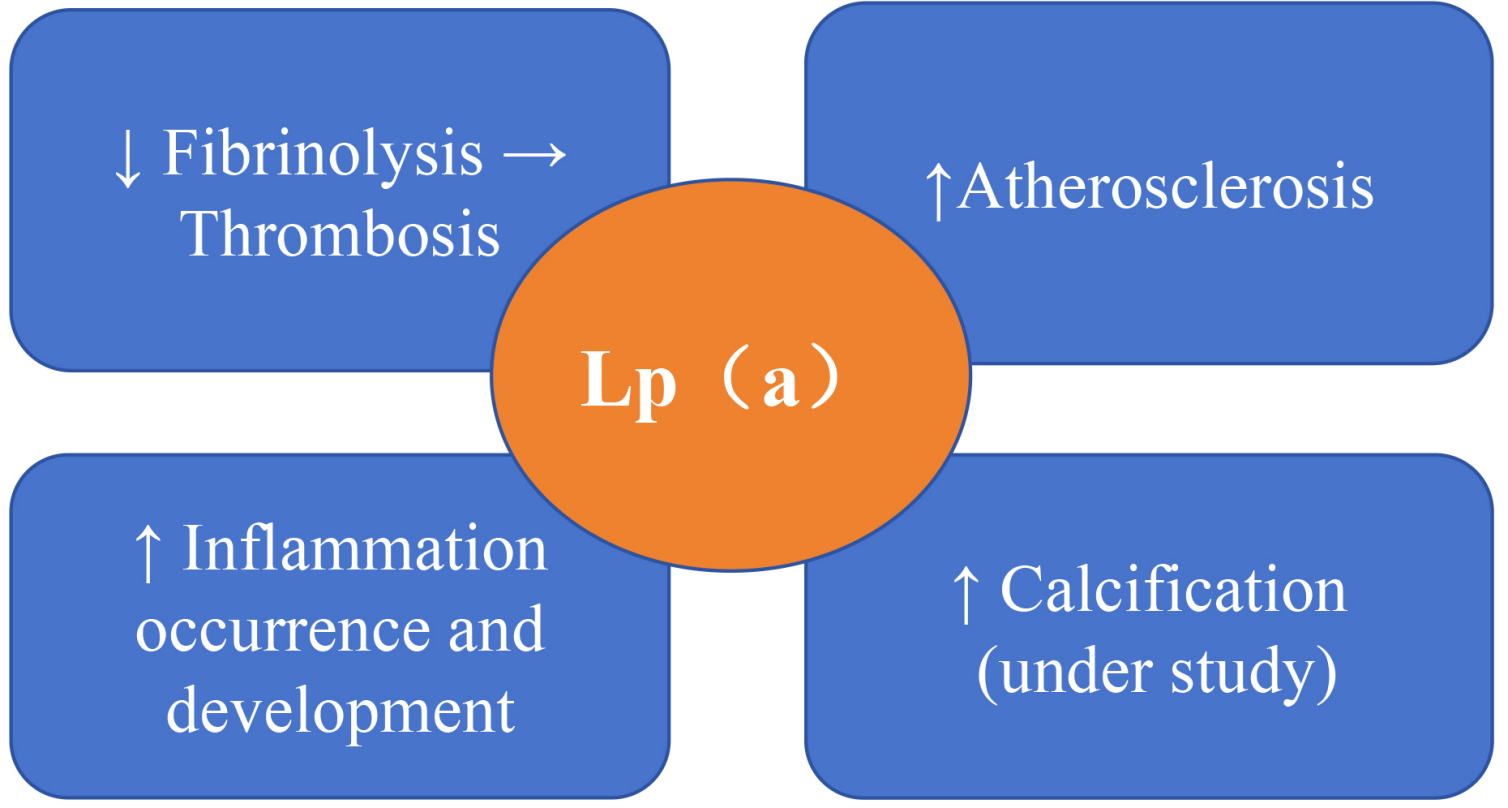
**Mechanisms by which lipoprotein (a) **(**Lp(a)) affects the prognosis of cardiovascular disease**. Lp(a) can increase cardiovascular risk by inducing vascular inflammation, atherosclerosis, calcification, and thrombosis, acting as a novel biomarker for cardiovascular disease (CVD) [20, 21, 22, 23]. The upward arrow indicates an increase, the downward arrow indicates a decrease, and the rightward arrow indicates that the reduction in the fibrinolysis process leads to thrombosis.

In addition to the traditional risk factor LDL-C, Lp(a) has been confirmed as an independent risk factor for all-cause mortality and MACE in post-PCI patients [18, 19]. Lp(a) is a complex formed by one molecule of apolipoprotein (a) and one molecule of LDL-C through covalent bonds. As shown in Fig. 1, Lp(a) can significantly increase cardiovascular risk by inducing vascular inflammation, promoting atherosclerosis, calcification, and thrombosis [20, 21, 22, 23]. In 2018, the American Heart Association (AHA) suggested that Lp(a) ≥125 nmol/L (≥50 mg/dL) should be regarded as an independent risk factor for atherosclerotic cardiovascular disease (ASCVD) [21, 24]. Zhang *et al*. [19] further confirmed that Lp(a) levels are independent predictors of MACE events within 1 year after PCI in CAD patients. Moreover, Zhang *et al*. [25] found that elevated Lp(a) levels are closely related to long-term adverse prognosis in patients with in-stent restenosis (ISR) after PCI. These findings suggest that measuring Lp(a) levels can aid in risk stratification for post-PCI. However, previous studies have employed heterogeneous assays for Lp(a) quantification. To mitigate the limitations of concentration measurement, current guidelines recommend the preferential use of a standardized particle concentration assay (nmol/L), thereby enhancing inter-study comparability and clinical concordance [26, 27].

### 2.4 High-Density Lipoprotein-Related Indicators

The pathological basis of cardiovascular diseases (CVD) is inflammation and lipid metabolism abnormalities. Compared to other indicators, the monocyte-to-high-density lipoprotein ratio (MHR) and the neutrophil-to-high-density lipoprotein ratio (NHR) can more comprehensively reflect the patient’s inflammatory state and lipid metabolism [28, 29, 30]. Studies have shown that the severity of CAD is positively correlated with MHR and NHR [21], which are more accurate prognostic indicators for post-PCI patients. For example, Yu *et al*. [31] demonstrated that MHR in ACS patients after PCI is positively correlated with the Gensini score. It can serve as an independent predictor of in-hospital MACE events. Meanwhile, the sensitivity and specificity of NHR for predicting adverse events related to ACS after PCI are as high as 77.6% and 74.2% [32, 33]. Previous studies have shown that MHR and NHR can be used for risk stratification of post-PCI CAD patients and for predicting short-term and long-term prognosis. However, how to quantitatively assess their utility remains an important direction for future research. Single-center designs and abbreviated follow-up periods may introduce heterogeneity in the distribution of clinical parameters across diverse geographic and ethnic populations. Therefore, large-scale, multicenter studies with extended follow-up are needed to enhance the generalizability and clinical applicability of lipid-related biomarkers.

## 3. Glucose and Related Metabolic Indicators

### 3.1 IR and CAD

**Fig. 2.**
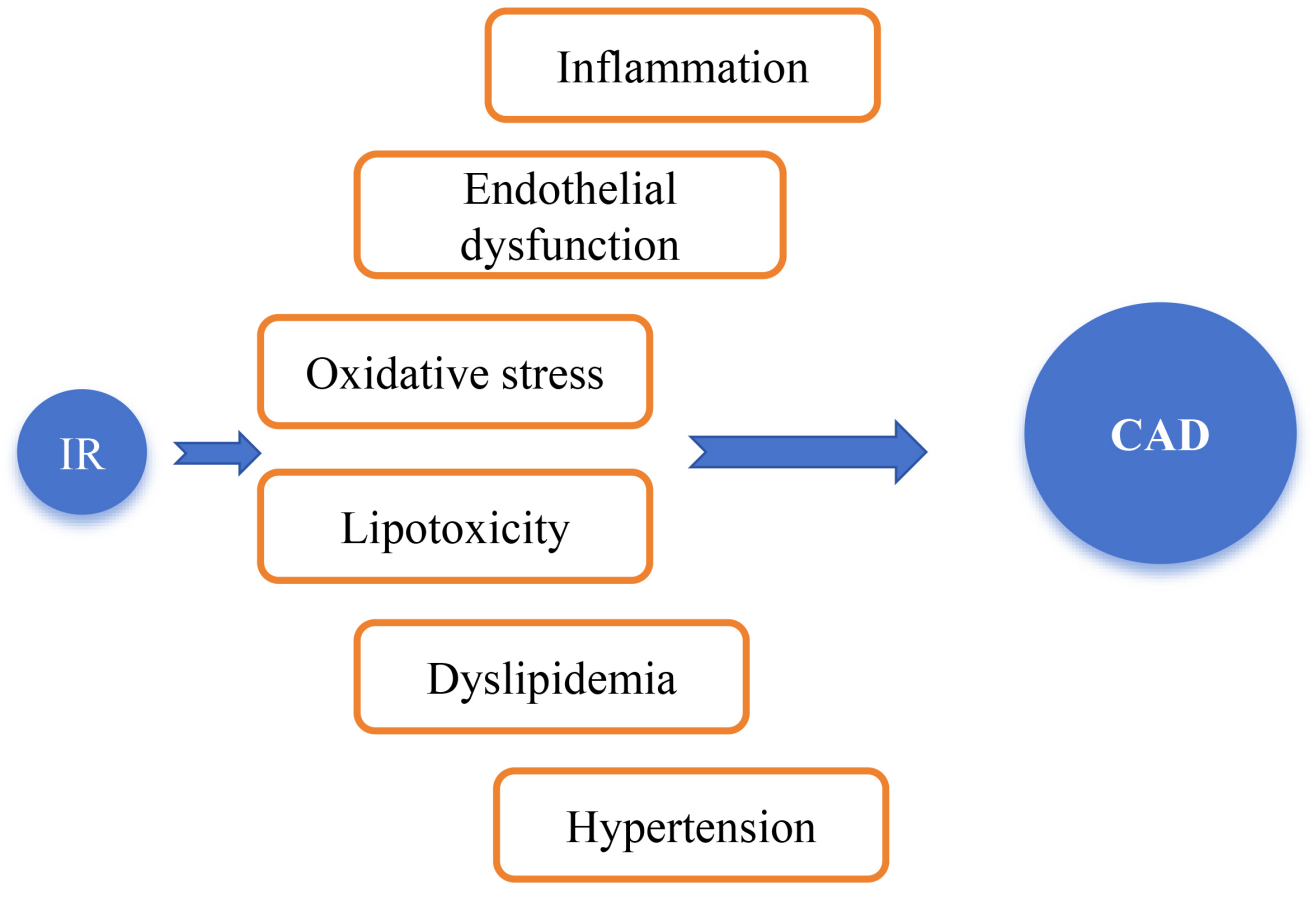
**Mechanisms by which insulin resistance (IR) leads to the occurrence and development of coronary artery disease (CAD)**. IR was identified many years ago as a key mediator of metabolic disorders, type 2 diabetes mellitus (T2DM), and CVD. IR can accelerate the progression of CAD through mechanisms such as inflammatory responses, oxidative stress, dyslipidemia, hypertension, and endothelial dysfunction [34].

As shown in Fig. 2, IR can accelerate the progression of CAD through mechanisms such as inflammation, oxidative stress, dyslipidemia, and endothelial dysfunction [34]. Although the “hyperinsulinemic-euglycemic clamp” is the “gold standard” for measuring IR, its complexity limits its clinical application [35]. In recent years, studies have found that the triglyceride-glucose index (TyG index = ln [fasting triglycerides (mg/dL) × fasting plasma glucose (mg/dL) / 2]) is superior to the homeostasis model assessment of insulin resistance (HOMA-IR) in evaluating IR. It has been established as a reliable alternative indicator for IR, and provides a cost-effective and reproducible alternative for large-scale epidemiological and clinical investigations [36, 37].

### 3.2 TyG Index

The TyG index is easily obtainable and is a better cardiovascular risk predictor than fasting plasma glucose (FPG) and glycated hemoglobin (HbA1c) [38]. It can effectively predict the long-term prognosis of CAD patients after PCI [39]. Multiple studies have confirmed that the TyG index performs significantly in predicting recurrent adverse cardiovascular events in ACS patients [40, 41]. Chen *et al*. [39] first demonstrated that patients with a higher TyG index (≥9.28 mg/dL) are more likely to require revascularization after PCI. Cheng *et al*. [42] further pointed out that elevated TyG index levels after PCI are positively correlated with ISR. These studies indicate that the TyG index is an independent predictor of adverse cardiovascular outcomes in post-PCI CAD patients [43]. It can provide important references for doctors to develop personalized treatment and prevention strategies [44]. However, further studies have found that compared with the TyG index alone, some improved TyG index-related indicators can significantly enhance the effectiveness of IR assessment [45] and are closely related to the progression of coronary atherosclerosis [46].

### 3.3 Improved TyG Index-Related Indicators

Combinations of the TyG index with obesity indicators (such as triglyceride-glucose-body mass index ratio (TyG-BMI = ln [Fasting TG (mg/dL) × FPG (mg/dL) / 2] × BMI (kg/m^2^)), triglyceride glucose-waist-to-height ratio (TyG-WHtR = ln [Fasting TG (mg/dL) × FPG (mg/dL) / 2] × Waist Circumference (cm) / Height (cm)), and triglyceride glucose-waist circumference index (TyG-WC = ln [Fasting TG (mg/dL) × FPG (mg/dL) / 2] × WC (cm)) have recently been considered more effective indicators for assessing IR [47]. For example, Xia *et al*. [48] showed that TyG-WC and TyG-WHtR perform better than the TyG index in predicting ASCVD events. Additionally, Cheng *et al*. [42] demonstrated that TyG-BMI is proportionally related to the incidence of major adverse cardiovascular and cerebrovascular events (MACCE) in elderly and female CAD patients after stent implantation. Compared with the TyG index alone, TyG-BMI, TyG-WC, and TyG-WHtR incorporate body-fat distribution parameters. This integration markedly enhances the predictive accuracy for both short and long-term MACE and all-cause mortality in patients with CAD after PCI. These composite indices exhibit differential advantages across age-stratified subgroups: TyG-WC, reflecting central adiposity, demonstrates heightened sensitivity in individuals aged <65 years, whereas TyG-WHtR, which adjusts for stature, confers pronounced incremental value in younger patients and in those without comorbidities [49, 50].

Cumulative TyG exposure is significantly associated with MACE, all-cause mortality, and ISR following PCI [51, 52]. A prospective cohort study further demonstrated that greater cumulative TyG exposure is linked to an increased risk of post-PCI MACE [53]. Considering the advantages of the improved TyG index-related indicators in assessing IR, future research should explore their application value in post-PCI patients. This will provide more accurate risk assessment tools for clinical use.

### 3.4 Stress Hyperglycemia Rate (SHR) and Fasting Blood Glucose to High-Density Lipoprotein Cholesterol Ratio (GHR)

The SHR assesses the state of relative hyperglycemia by comparing admission blood glucose levels with the average glucose levels estimated from HbA1c. It has been reported that SHR should be regarded as a high-risk prognostic indicator for patients with ST-segment elevation myocardial infarction (STEMI) following PCI. A study has shown that in patients with STEMI undergoing PCI, SHR is significantly associated with increased in-hospital mortality and all-cause mortality risk, regardless of whether the patient has diabetes [54]. A multicenter observational study further demonstrated that each tertile increase in SHR was associated with a 28% rise in 30-day MACE risk. This metric independently predicted prognosis after PCI in STEMI patients [55]. Additionally, it has been proven that GHR can independently predict the risk of adverse outcomes in post-PCI CAD patients without diabetes [56]. To date, studies examining the prognostic utility of the SHR and the GHR specifically in patients after PCI are scarce. Existing evidence is primarily derived from populations in intensive care units or general medical wards. Data for older adults, women, and individuals with comorbid anxiety or depression remain limited.

## 4. Nutrition-Related Metabolic Indicators

### 4.1 Serum Albumin

**Fig. 3.**
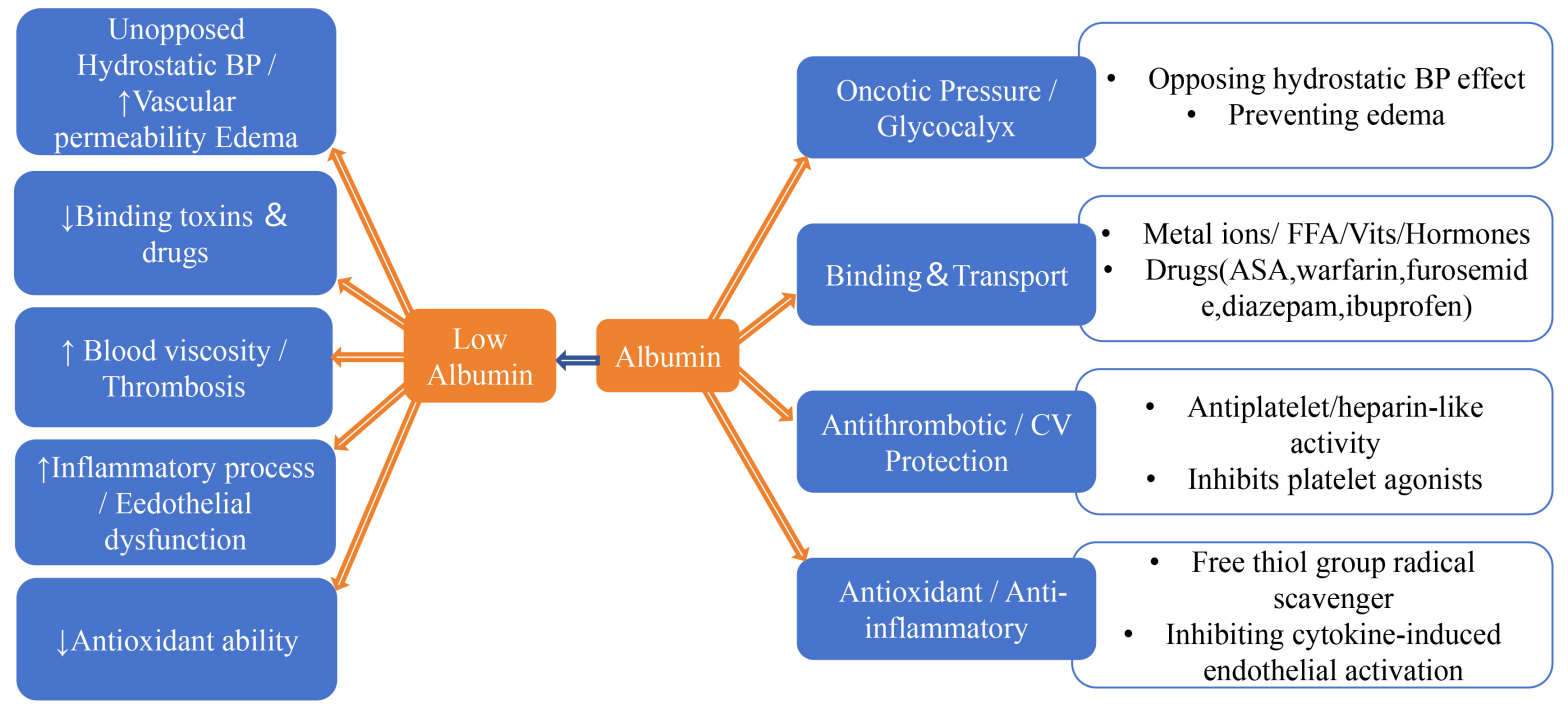
**Mechanisms by which albumin contributes to the occurrence and development of CVD and the impact of decreased albumin levels**. Low levels of serum albumin (SA) can accelerate the occurrence and development of CVD through the following mechanisms: (1) Refractory hypertension/vessel permeability increase leading to edema; (2) Reduced binding to toxins and drugs; (3) Increased blood viscosity/thrombosis; (4) Accelerated inflammatory processes/endothelial dysfunction; (5) Decreased antioxidant capacity [9, 10, 57, 58, 59, 60, 61, 62, 63]. The upward arrow indicates an increase, the downward arrow indicates a decrease. CV, cardiovascular; BP, blood pressure; FFA, free fatty acids; ASA, aspirin; CVD, cardiovascular disease.

Albumin is the main protein in the human body, involved in a variety of important physiological functions. As shown in Fig. 3 (Ref. [9, 10, 57, 58, 59, 60, 61, 62, 63]), these include: (1) maintaining plasma oncotic pressure and capillary permeability; (2) acting as a carrier for many endogenous and exogenous substances, participating in their transport and distribution; (3) affecting the pharmacokinetics of many drugs, regulating the absorption, distribution, metabolism, and excretion of drugs; (4) inhibiting platelet aggregation, protecting endothelial function of blood vessels, and maintaining vascular homeostasis; (5) having anti-inflammatory and antioxidant effects, reducing oxidative stress and inflammatory reactions [9, 10, 57].

SA functions as a natural antiplatelet and anticoagulant, as well as the principal antioxidant in plasma. It also acts as a scavenger of inflammatory mediators, thereby exerting critical effects across the pathophysiology of CVD [58]. Experimental data indicate that reduced SA concentrations diminish platelet activation–aggregation inhibition by neutralizing adenosine 5^′^-diphosphate (ADP), thromboxane A_2_, and coagulation factors. This subsequently prevents glycoprotein IIb/IIIa complex (GPIIb/IIIa) activation and fibrinogen binding. Additionally, reduced SA levels amplify inflammatory and oxidative stress, decrease nitric oxide bioavailability, and ultimately lead to endothelial dysfunction [59, 60, 61, 62, 63]. Collectively, these alterations promote coronary plaque progression and thrombus formation.

It has been confirmed as an important risk factor for the progression of cardiovascular diseases and is also an economical, simple, and easily obtainable prognostic predictor [64]. In a prospective study involving 734 individuals, it was found that hypoalbuminemia (<3.5 g/dL) accelerates the progression of adverse outcomes in CAD patients [65]. Additionally, research by Shiyovich *et al*. [57] first revealed that a decline in albumin levels within the first year after PCI is a marker of long-term adverse prognosis. This finding further highlights the significance of SA in evaluating the prognosis of post-PCI patients.

### 4.2 Combination of SA With Other Prognostic Risk Markers

Studies have shown that when albumin is combined with other risk factors (such as C-reactive protein, neutrophils, alkaline phosphatase, etc.), its predictive value is significantly enhanced [58]. Results from a study involving 2164 patients showed that the ratio of low SA to high-sensitivity C-reactive protein (hs-CRP) (SA <4.1 g/dL and hs-CRP ≥0.10 mg/dL) is an independent risk factor for MACE events in CAD patients after PCI, demonstrating that low SA and high hs-CRP levels have a synergistic adverse effect on long-term MACE risk [66]. Additionally, it has been reported that the neutrophil-to-albumin ratio (NAR) is significantly associated with the severity of CAD and is superior to the NAR and systemic inflammatory-immune index (SII) in distinguishing the severity of coronary arteries [67]. A high level of alkaline phosphatase-to-albumin ratio (AAR) (>1.77) is an independent predictor of adverse prognosis in CAD patients after PCI [68]. In summary, existing studies have shown that the combined application of SA with other biological markers exhibits stronger efficacy in predicting the prognosis of post-PCI CAD patients.

### 4.3 Homocysteine (Hcy)

Elevated plasma levels of Hcy have been confirmed to be closely related to endothelial dysfunction, vascular inflammation, and the progression of atherosclerosis. Studies have shown that the risk of vascular disease is significantly associated with elevated Hcy levels. A study involving 39,242 participants demonstrated that patients with total homocysteine (tHcy) >15.3 mol/L have 4.35 times the risk of stroke, 3.4 times the risk of myocardial infarction, and 1.68 times the total mortality rate of CAD compared to individuals with tHcy <15.3 mol/L [69]. Moreover, high levels of Hcy (≥12 µmol/L) are independently associated with an increased risk of long-term cardiovascular events in post-PCI patients [70]. Therefore, further exploration is needed regarding the specific application value of Hcy as a prognostic predictor in post-PCI clinical outcomes. However, current evidence demonstrates that oral folic acid and vitamin B12 supplementation alone do not reduce cardiovascular events despite lowering Hcy levels [71]. Consequently, individualized interventional trials specifically in patients after PCI are warranted, and the precise utility of Hcy as a prognostic indicator in this population requires further investigation.

## 5. Kidney-Related Metabolic Indicators

### 5.1 Serum Uric Acid

**Fig. 4.**
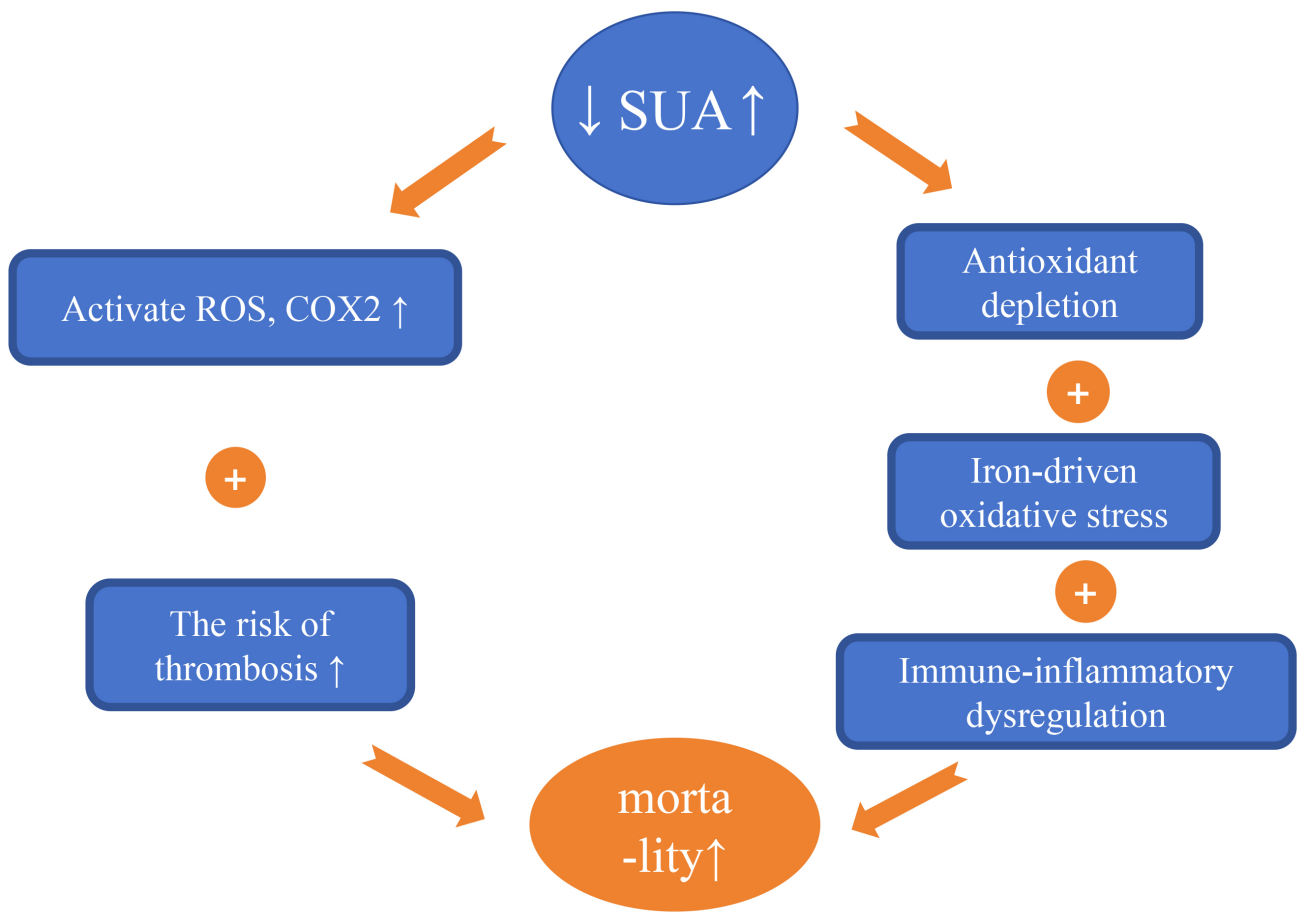
**Mechanisms by which fluctuations in serum uric acid (SUA) levels affect the occurrence and development of cardiovascular disease**. UA serves as an independent predictor of all-cause and cardiovascular mortality in patients with CAD. Elevated SUA levels can lead to: (1) Increased production of reactive oxygen species (ROS) and reactive nitrogen species (RNS), as well as the activation of cyclooxygenase 2, resulting in inflammatory stress. (2) Increased mortality associated with thrombotic diseases. Conversely, low levels of SUA can result in: (1) Antioxidant depletion. (2) Immune-inflammatory dysregulation. (3) Iron-catalyzed oxidative stress—collectively impairing endothelial integrity and elevating MACE risk in CAD [8, 73, 74, 75]. The upward arrow indicates an increase, the downward arrow indicates a decrease. CAD, coronary artery disease; UA, uric acid; MACE, major adverse cardiovascular events.

Numerous studies have shown that SUA is an independent predictor of all-cause and cardiovascular mortality in CAD patients [72]. As shown in Fig. 4 (Ref. [8, 73, 74, 75]), Elevated SUA levels can promote oxidative stress, inflammatory responses, and thrombotic risk. While low SUA levels impair endothelial integrity through three mechanisms: antioxidant depletion, immune-inflammatory dysregulation, and iron-catalyzed oxidative stress. Additionally, UA regulates T-cell activation and cytokine release, so low SUA may disrupt immune-inflammatory homeostasis, attenuate immune surveillance, and thereby impair endothelial repair and plaque stability.

For example, a study has shown that SUA has a U-shaped relationship with long-term all-cause mortality risk in CAD patients, with those in the optimal SUA range (5.59 mg/dL ≤ SUA < 6.8 mg/dL) likely having a better prognosis [74]. Further research has found that in STEMI patients undergoing PCI, elevated SUA levels are associated with higher mortality rates [76]. Hyperuricemia (>5.6 mg/dL) has been confirmed as an independent risk factor for MACE events in ACS and hypertensive patients after PCI, with a positive correlation between Gensini scores and uric acid levels in these patients [73]. Additionally, foreign scholars believe that in CAD patients undergoing PCI, high SUA levels (UA >7.94 mg/dL) are significantly associated with increased 10-year mortality [77]. Consistent with the above findings, a study based on the Clinical Depth Data Accumulation System (CLIDAS) showed that hyperuricemia is associated with an increased incidence of MACE in post-PCI patients [78]. However, whether treating hyperuricemia can reduce the incidence of cardiovascular events still needs further clinical trial verification.

### 5.2 Combination of SUA With Other Prognostic Risk Markers

In recent years, the serum uric acid to creatinine ratio (SUA/SCr) ratio has emerged as a novel biomarker reflecting endogenous uric acid levels, gaining considerable attention for its ability to mitigate the confounding effects of estimated glomerular filtration rate (eGFR) variability on SUA. Existing research has shown its association with the occurrence risk and mortality of metabolic syndrome [79]. A growing body of evidence has established that the SUA/SCr ratio surpasses SUA alone in predicting cardiovascular events. However, no large-scale, dedicated studies have yet investigated the prognostic implications of the SUA/SCr ratio in patients with CAD following PCI.

## 6. Conclusions

Evidence indicates that post-procedural outcomes in patients with CAD following PCI are influenced by multiple factors. These include lipid status, glycaemic control, nutritional state, renal function, and chronic low-grade inflammation. Elevated levels of Lp(a), MHR, NHR, TyG index, SUA, and low levels of SA are all independent risk predictors. In particular, combined indicators such as TyG-BMI, TyG-WC, TyG-WHtR, NAR, and AAR have shown stronger prognostic predictive capabilities. Moreover, long-term exposure to adverse metabolic states, such as persistently elevated LDL-C or TyG index, is also associated with higher cardiovascular risks. The present study proposes using routine laboratory parameters to predict long-term risk, thereby advancing the decision window. We recommend integrating these four indices into a risk-assessment table. This tool can be applied at hospital discharge and during outpatient follow-up visits to enable dynamic prognostic evaluation and guide tailored therapeutic adjustments.

Whereas the Synergy between Percutaneous Coronary Intervention with Taxus and Cardiac Surgery (SYNTAX) score primarily quantifies lesion complexity to guide PCI strategy, the multiparametric metabolic prediction models outlined herein are intended for post-procedural risk management during longitudinal follow-up. Existing conclusions are largely derived from single-center retrospective cohorts within specific populations. Therefore, their generalizability and clinical value must be validated in more diverse populations. Future studies should employ harmonized, standardized assays alongside multi-ethnic, large-scale cohorts and establish ethnicity-specific cut-off values to minimize confounding attributable to potential racial differences in metabolic biomarkers. It is anticipated that future iterations of novel predictive models will exhibit enhanced discriminative accuracy and prognostic utility for patients following PCI.

## Abbreviations

CVD, cardiovascular disease; ASCVD, atherosclerotic cardiovascular disease; PCI, percutaneous coronary intervention; CAD, coronary artery disease; MACE, major adverse cardiovascular events; CHD, coronary heart disease; LDL-C, low-density lipoprotein cholesterol; TG, increased triglyceride; HDL-C, high-density lipoprotein cholesterol; Lp(a), lipoprotein (a); MHR, monocyte-to-high-density lipoprotein ratio; NHR, neutrophil-to-high-density lipoprotein ratio; IR, insulin resistance; TyG, triglyceride-glucose index; TyG-BMI, triglyceride-glucose-body mass index ratio; TyG-WHtR, triglyceride-glucose-waist-to-height ratio; TyG-WC, triglyceride-glucose-waist circumference ratio; hs-CRP, high-sensitivity C-reactive protein; SHR, stress hyperglycemia rate; GHR, glucose-to-high-density lipoprotein cholesterol ratio; SA, serum albumin; NAR, neutrophil-to-serum albumin ratio; AAR, alkaline phosphatase to serum albumin ratio; Hcy, homocysteine; tHcy, total Homocysteine; SUA, serum uric acid; SUA/SCr, serum uric acid to creatinine ratio; ISR, in-stent restenosis; T2DM, type 2 diabetes mellitus; FPG, fasting plasma glucose; HbA1c, glycated hemoglobin; CLIDAS, Clinical Depth Data Accumulation System; STEMI, ST-segment elevation myocardial infarction; SII, systemic inflammatory-immune index; ROS, reactive oxygen species; RNS, reactive nitrogen species; AHA, American Heart Association; OCT, optical coherence tomography; ADP, adenosine 5^′^-diphosphate; GPIIb/IIIa, glycoprotein IIb/IIIa complex.
